# Epidemiology and Incidence of Retinoblastoma in the Middle East: A Nationwide Study in Lebanon

**DOI:** 10.7759/cureus.18696

**Published:** 2021-10-12

**Authors:** Said El Hage, Elyas Wakim, Lea Daou, Jad El Masri, Pascale Salameh

**Affiliations:** 1 General Medicine, Lebanese University, Beirut, LBN; 2 Faculty of Medical Sciences, Lebanese University, Beirut, LBN; 3 Neurosciences Research Center, Lebanese University, Beirut, LBN; 4 Public Health, National Institute of Public Health, Clinical Epidemiology and Toxicology (INSPECT-LB), Beirut, LBN; 5 Public Health, University of Nicosia Medical School, Nicosia, CYP; 6 Pharmacy, Lebanese University, Beirut, LBN

**Keywords:** ophthalmology, cancer, incidence, middle east, lebanon, retinoblastoma

## Abstract

Background: Retinoblastoma, although a rare pediatric cancer, can lead to disastrous outcomes if not managed early. This mishap often happens in developing countries. Conversely, early diagnosis and treatment of retinoblastoma in developed countries were associated with a surge in RB1 gene carriers.

Aim: The authors aimed to evaluate the incidence of retinoblastoma in the Lebanese population aged between 0 and 19 years according to age, sex, and other variables and compare the age-standardized incidence rates with regional and worldwide countries.

Methods: Data were retrieved from the National Cancer Registry (NCR) of the Ministry of Public Health (MOPH). Subsequently, incidence rates, age-standardized rates, and fictional incidence rates excluding the emigrant population were calculated. Retinoblastoma trends were found using the Joinpoint regression program software.

Results: From the 38 cases of retinoblastoma recorded in the nine years cohort, 29 cases occurred in the 0-4 age group, and age-standardized rates were 2.8 and 3.6 per million person-years for the 0-19 and 0-14 age groups, respectively. When the refugee population was excluded, the ASR 0-19 almost doubled from 2.8 per million person-years to 5.16 per million person-years. Joinpoint regression revealed that retinoblastoma trends were divided into two segments showing a decrease from 2005 until 2011 and a rising trend in 2011-2015. When compared to other countries in the region, Lebanon had low-intermediate 0-19 ASRs.

Conclusion: Retinoblastoma incidence in Lebanon is in the lower margin of the worldwide average and could be underestimated due to the underdiagnosis in the refugee population. Efforts are being deployed to overcome the financial barriers in the treatment of retinoblastoma by coordinating with neighboring Arab countries.

## Introduction

Retinoblastoma is the most common ocular cancer in the pediatric population. It arises from multipotent retinal cell precursors when both homologous loci of the RB1 tumor suppressor gene become nonfunctional. The second mutation is always somatic, but the first mutation might be either somatic or germline, leading to either sporadic or hereditary retinoblastoma, respectively. Patients who have received a germline RB1 mutation have the nonfunctional allele in all their cells and are thus prone to developing malignancies in multiple other organs with increasing age. They might also transmit the mutation to their progeny. However, it is noteworthy that only 10% of children with retinoblastoma have a positive family history [1].

Despite being the most frequent neoplasm of the eye in childhood, retinoblastoma accounts for no more than 2% of all pediatric cancers [2] and is a rare intraocular malignancy compared to uveal melanoma and choroidal metastasis [3]. Worldwide the incidence of retinoblastoma is estimated to be around 11 cases per million children younger than five years of age, or one case of retinoblastoma per 18000-30000 live births, with some countries claiming more than one case of retinoblastoma per 18000 live births, such as the Philippines [4]. 

In a recent publication, Stacey et al. pointed to an increase in retinoblastoma cases in Europe. This finding was attributed to the upsurge in the number of carriers of the RB1 gene in the population since, in these high-income countries, retinoblastoma diagnosis occurs in an early stage that doesn’t require enucleation, avoiding disfigurement and death and thus toning down the coefficient of selection. Children of these individuals have a 50% chance of developing this autosomal dominant disease [5].

While mortality from retinoblastoma is estimated to be fewer than 5% in high-income countries, it can reach up to 70% in African countries because retinoblastoma is not recognized and treated on time [6], denoting a large gap between countries. Lebanon, a third-world country in the Middle East region but renowned for its quality of care, was ranked 23^rd^ worldwide using Bloomberg’s Healthcare Efficiency Index as of 2019 [7]. In this country, no study has tackled the epidemiology of retinoblastoma; therefore, it is unknown whether Lebanon follows the same trends in the surge of cases as in high-income countries.

This study aims to evaluate the incidence of retinoblastoma in Lebanese children and adolescents (0 to 19 years old) according to age, sex, and other variables and compare the age-standardized incidence rates with regional and worldwide countries.

## Materials and methods

Data and ethical consideration

Retinoblastoma data were retrieved from the National Cancer Registry (NCR) of the Ministry of Public Health (MOPH) [7]. Since the NCR tables are available publicly, an Institutional Review Board approval was not necessary. Data consisted of pediatric cancer cases from 0 to 19 years of age across the entire country from 2005 till 2012 and 2015. The International Incidence of Childhood Cancer (ICCC) provided gender data from 2008 till 2010 publicly on their website [8]. The ICCC is a cooperative project between the well-known International Agency for Research on Cancer (IARC) and the International Association of Cancer Registries (IACR). These agencies seek to propagate high-quality cancer data in the pediatric population [8].

Incidence rates calculations, standardization, and comparison

An incidence rate per year is the number of cases diagnosed in one year divided by the corresponding mid-year population. Incidence rates were calculated using Lebanon’s population from the World Prospect Population (WPP), which included the five-year age groups and gender population. Therefore, age-specific incidence rates were calculated for the 0-4, 5-9, 10-14, and 15-19 age groups, along with the total incidence rate (crude rate) [9]. Rates were presented by million person-years. They were implemented through a standardization procedure, which assumes equal age group distribution between all countries, improving data comparability. Analogous to the standardized rates used in the ICCC, we used the Segi et al. (1950) world standard population as a reference population [10]. Both the 0-14 and 0-19 age-standardized rates were calculated. Results were then compared to other countries using the ICCC database. Regional Arab and Middle Eastern countries and other worldwide countries were included in the comparison of incidence rates.

The fictional population

Lebanese population statistics are scarce due to political, ethnic, religious, and social hurdles. In addition, it is estimated that Lebanon hosts one of the largest refugee populations in the world, with around 500,000 Palestinians and 1,500,000 Syrian refugees. Due to the unavailability of the NCR’s population demographic factors, we introduced a new metric to calculate incidence rates in Lebanon. This metric is based on a “fictional” population: the Lebanese population excluding Syrian and Palestinian refugees, obtained by the MOPH Statistical Bulletin, to provide a contextual analysis in Lebanon. Using this fictional population, the resulting fictional incidence rates were calculated. In fact, if the refugee population was underdiagnosed, retinoblastoma incidence rates using the fictional population could help assess the margin of incidence rates within the Lebanese population itself.

Trend dynamics

To address the trends of retinoblastoma in the Lebanese population, we used the Joinpoint regression program software. This program is supported by the National Cancer Institute (NCI) and used for cancer trends analysis [11]. Effectively, this software plots the cancer rates on the y-axis, with the years on the x-axis. It also gives the Annual Percentage Changes (APC) of retinoblastoma. An additional feature is its ability to do a “piecewise” analysis by dividing trends into segments. The segments are separated by “Joinpoints,” which mark an abrupt change in the incidence rate trends. In this study, a p-value of less than 0.05 was considered to be significant.

## Results

Retinoblastoma rank and number of cases

**Figure 1:**
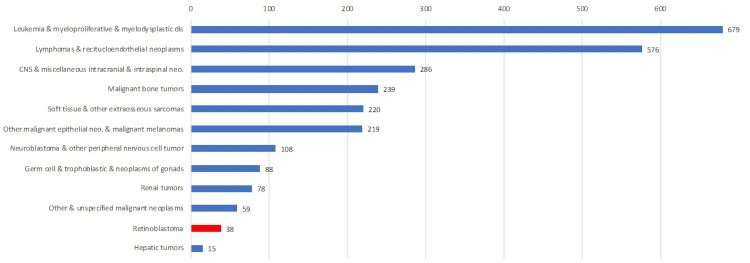
Ranks of ICCC childhood cancer by cases in Lebanon from 2005-2012; 2015 (original figure)

Among the 12 ICCC categories, retinoblastoma ranked modestly as the 11^th^ category of childhood cancers, with an aggregate of 38 cases in the nine years of the cohort. Retinoblastoma was only followed by hepatic tumors, which accounted for just 15 cases in total, the latter being the rarest type of childhood cancers (Figure 1). Most of the retinoblastoma cases appeared in the earliest age groups, with 29 out of 38 cases occurring in the 0-4 age group (Table 1). Interestingly, no cases of retinoblastoma were recorded in the 15-19 age group.

**Table 1:** Number of cases of retinoblastoma in Lebanon from 2005-2012; 2015

Years	0-4y	5-9y	10-14y	15-19y	0-14y	0-19y
2005	3	1	0	0	4	4
2006	1	0	1	0	2	2
2007	6	0	1	0	7	7
2008	1	1	0	0	2	2
2009	1	1	0	0	2	2
2010	6	1	0	0	7	7
2011	0	0	0	0	0	0
2012	6	0	0	0	6	6
2015	5	3	0	0	8	8
TOTAL	29	7	2	0	38	38

Age-adjusted rates and age-standardized rates

Age-adjusted rates varied through the studied years from null rates in 2011 and reached 16.18 per million in 2010 in the 0-4 age group. The 5-9 and 10-14 age groups had lower age-adjusted rates than the 0-4 age group, reaching a maximum incidence rate of 5.17 per million in 2015 and 1.97 per million in 2007, respectively (Table 2). Results also showed that the total age-standardized rates (ASR) were 2.8 and 3.6 per million person-years for the 0-19 and 0-14 age groups, respectively. Besides, a fictional rate was utilized, using the Lebanese population as the denominator, excluding Syrian and Palestinian refugees. These rates are used for comparative and contextualization purposes only since underdiagnosis in the refugee population is prevalent. The ASR 0-19 almost doubled from 2.8 per million person-years in the Lebanese population to 5.16 per million person-years when excluding refugees (Table 2).

**Table 2:** Age-adjusted and age-standardized rates (ASR) per million person-years of retinoblastoma in children and adolescents (0-19 years) in Lebanon, between 2005-2012; 2015 * Using Lebanese population without Syrian/Palestinian refugees

Year	0-4y	5-9y	10-14y	15-19y	0-19y	0-14	ASR 0-19	ASR 0-14
2005	7.05	2.06	0.00	0.00	2.12	2.81	2.63	3.40
2006	2.42	0.00	1.94	0.00	1.07	1.42	1.16	1.50
2007	15.26	0.00	1.97	0.00	3.80	5.15	5.02	6.48
2008	2.70	2.26	0.00	0.00	1.11	1.53	1.38	1.78
2009	2.80	2.33	0.00	0.00	1.13	2.09	1.57	1.83
2010	16.18	2.36	0.00	0.00	3.91	5.47	5.45	7.03
2011	0.00	0.00	0.00	0.00	0.00	0.00	0.00	0.00
2012	13.12	0.00	0.00	0.00	3.00	4.14	3.94	5.08
2015	8.15	5.17	0.00	0.00	3.35	4.48	3.74	4.82
TOTAL	7.62	1.66	0.43	0.00	2.21	3.01	2.80	3.61
Total, Fictional*	11.02	2.27	0.57	0.00	2.98	4.12	5.16	4.00

Gender distribution

Available gender data from 2008 till 2010 revealed higher retinoblastoma cases in females than males (M/F ratio = 0.57). Effectively, results showed similar age-specific rates in the 0-4 age group (3.64 per million person-years). Males were not found to have retinoblastoma cases in the subsequent age groups. However, females had an age-specific rate of 2.32 per million person-years in the 5-9 age group (Table 3). 

**Table 3:** Age-adjusted and age-standardized rates (ASR) of retinoblastoma in males and females between 2008-2010, Lebanon

Rates	Male	Female
0-4y	3.64	3.64
5-9y	0.00	2.32
10-14y	0.00	0.00
15-19y	0.00	0.00
0-19y	0.75	1.31
0-14y	1.04	1.81
ASR 0-19	1.09	1.67
ASR 0-14	1.41	2.16
Ratio M/F 0-19	0.57	
Ratio M/F 0-14	0.33	

Cancer trends dynamics

**Figure 2:**
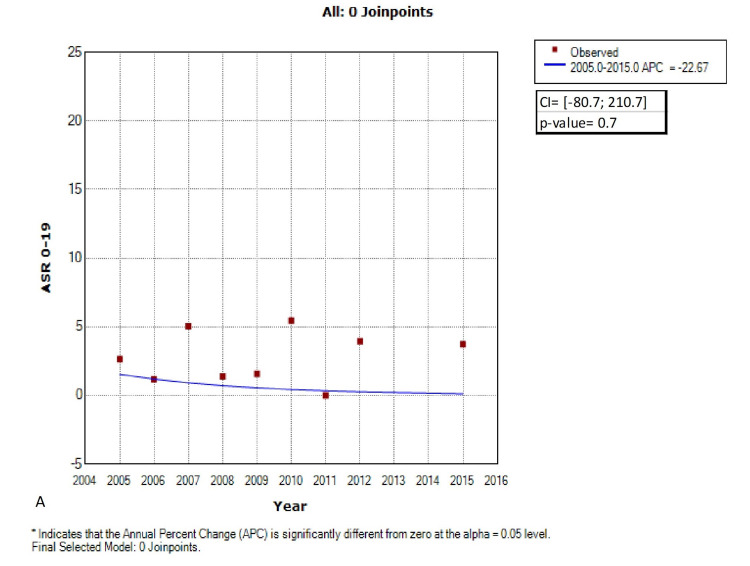
Age-standardized rates (ASR 0-19y) trends of retinoblastoma in children/adolescents between 2005-2012; 2015, Lebanon using 0 Joinpoint (original figure)

**Figure 3:**
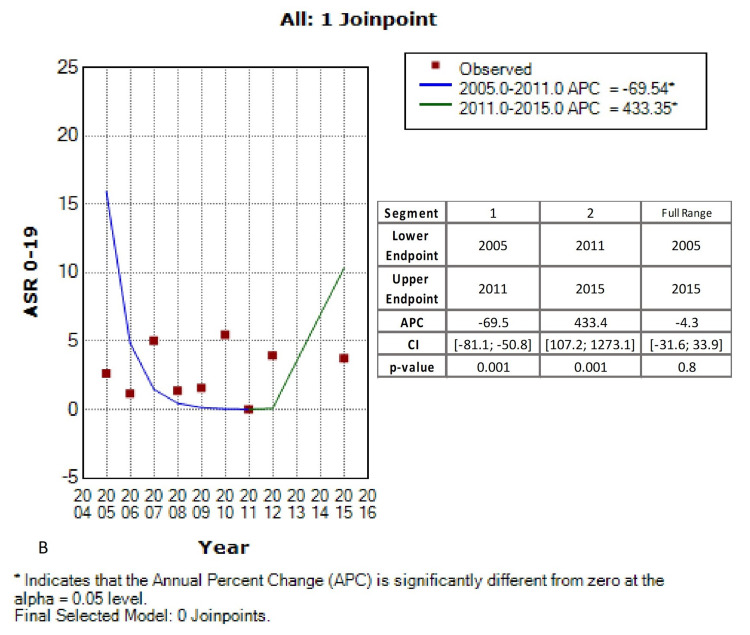
Age-standardized rates (ASR 0-19y) trends of retinoblastoma in children/adolescents between 2005-2012; 2015, Lebanon Using 1 Joinpoint (original figure)

Joinpoint regression, using piecewise analysis of the 0-19 ASRs across the years, revealed one joinpoint located in 2011. When using no joinpoint, the annual percentage change (APC) was -22.67% ([CI]: -80.7; 210.7), which means that retinoblastoma cases were decreasing at a rate of 22.67% per year. However, this decreasing trend was not statistically significant (p-value= 0.7) (Figure 2). Nonetheless, when accounting for the joinpoint found in 2011, retinoblastoma trends were divided into two segments. The first segment ranged from 2005 till 2011 and revealed a significant decline in retinoblastoma cases (APC: -69.5, CI: [-81.5; -50.8], p-value<0.001). Remarkably, an abrupt significant rising trend followed the decline, in the 2011-2015 range (APC: 433.4; CI: [107.2; 1273.1]; p-value<0.001) (Figure 3).

Comparison with other countries

When comparing with regional and worldwide countries, Lebanon fostered intermediate ASRs and age-specific rates. Lebanon presented a 0-19 ASR of 2.8 per million person-years. In regional countries, the lowest 0-19 ASR was found in Qatar (0.3 per million person-years) and the highest one in Jordan (5.5 per million person-years). Other random countries resulted in 0-19 ASRs ranging from 2.2 per million person-years in Bulgaria to 22.2 per million person-years in Mali (Table 4).

**Table 4:** Comparison of age-specific/adjusted rates and age-standardized rates (ASR) of Lebanon with other regional and random worldwide countries. * Gender data was only collected from 2008-2010

			ASR 0-19			Age specific rates per million			
Countries	Years	N	All	Male	Female	0-4	5-9	10-14	15-19
Regional countries									
Algeria (5 registries)	1996-2014	28	1.4	0.9	2	3.8	0.9	0	0.1
Bahrain	1998-2012	7	1.8	2.1	1.6	3.7	1.9	0	1.1
Egypt, Al Gharbiyah	1999-2010	27	1.7	1.4	2	5.5	0.2	0	0
Iran, Golestan	2004-2011	2	0.5	0.5	0.5	1.8	0	0	0
Israel (Jews and non Jews)	1990-2012	151	3	3.1	3	9.5	0.7	0.1	0
Jordan	2000-2012	174	5.5	6.3	4.8	16.9	1.7	0	0.1
Kuwait (Kuwait and non Kuwaitis)	1994-2012	14	0.9	1.1	0.7	1.6	0.5	1.4	0
Lebanon	2005-2012,2015*	38	2.8	1.09	1.67	7.62	1.66	0.43	0.00
Libya, Benghazi	2003-2008	14	4.1	4.5	3.6	11.8	2.1	0	0
Mauritius	2001-2013	10	2.6	1	4.2	8.6	0	0	0
Morocco (2 registries)	2005-2012	72	7.1	7.5	10.7	21.8	2.1	0.3	0
Qatar	2002-2014	1	0.3	0.5	0	0.9	0	0	0
Saudi Arabia, Riyadh	1994-2012	211	5.2	4.9	5.6	16.6	0.7	0.3	0
Tunisia (2 registries)	1993-2007	55	2.8	3.1	2.3	8.2	1.1	0	0
Turkey (8 registries)	1992-2012	138	3.2	3.4	3.1	10	0.8	0.1	0.1
Random Countries									
Argentina, paediatric	2000-2013	-	-	-	-	11.8	1	0.1	-
Australia	1992-2014	351	3.6	4.1	3.1	11.5	0.5	0	0
Belarus, paediatric	1990-2014	150	3.3	3.1	3.5	10.5	0.5	0	0.2
Bulgaria	1990-2013	68	2.2	2.4	1.9	6.8	0.5	0	0
Canada (9 registries)	1992-2013	464	3.7	3.8	3.7	12	0.4	0.1	
Chile, paediatric	200-2011	-	-	-	-	11.9	1.3	0	0
China (6 registries)	1990-2013	248	4.5	4.8	4	14.4	0.5	0	-
Cuba	2000-2012	91	3.1	2.8	3.4	9.3	0.6	0.7	-
Czech Republic	1990-2012	169	3.3	3.4	3.3	10.9	0.2	0.1	0.1
France, paediatric	2000-2012	-	-	-	-	12.6	0.5	0	-
Germany, paediatric	1996-2012	-	-	-	-	10.2	0.4	0.1	0
India (7 registries)	1990-2013	600	3.3	3.8	2.8	9.8	9.4	0.1	0
Italy (26 registries)	1992-2013	157	3.4	3.3	3.6	11.2	0.1	0.1	0
Japan (8 registries)	1990-2013	252	3.9	3.9	3.9	12.6	0.6	0	0
Kenya (2 registries)	2000-2012	78	5.4	6.4	4.5	16	2.3	0.3	-
Mali, Bamako	2005-2014	208	22.2	23.2	21.3	58.3	15.1	4.3	-
Pakistan, Lahore	2008-2012	82	4	4.4	3.6	11.7	1.7	0	0.1
South Africa, paediatric	1998-2012	-	-	-	-	7.7	0.8	0.1	0
Spain (7 registries)	1990-2013	107	3.1	3.4	2.9	10.1	0.4	0.1	0
UK	2000-2011	512	3.5	3.5	3.5	11.3	0.5	0.1	0
Uruguay	1993-2012	69	4.1	4.8	3.4	12.6	1.3	0	0
USA	1998-2012	3743	3.8	3.8	3.8	12.2	0.4	0	0

## Discussion

From the 38 cases of retinoblastoma recorded in the nine years cohort, 29 cases occurred in the 0-4 age group, and age-standardized rates were 2.8 and 3.6 per million person-years for the 0-19 and 0-14 age groups, respectively. Interestingly, when the refugee population was excluded, the ASR 0-19 almost doubled from 2.8 per million person-years to 5.16 per million person-years. Results showed similar age-specific rates in the 0-4 age group for both genders. Using joinpoint regression, retinoblastoma trends were divided into two segments showing a decrease from 2005 until 2011 and a rising trend in the 2011-2015 range. When compared to other countries in the region, Lebanon had low-intermediate 0-19 ASRs.

Retinoblastoma in the Lebanese context

When compared to other countries, Lebanon is a mosaic country in terms of disease distribution, with increasing chronic systemic diseases such as hypertension, diabetes mellitus, and cardiac diseases which are common in high-income countries [12], and high, yet declining, infectious diseases common to third world countries, with some communicable diseases such as hepatitis A spreading in the population as outbreaks in the era of refugees [13]. Lebanon is also notable for the high percentage of consanguineous marriage, accounting for 35.5% of total marriages in this country [14] and further contributing to the spread of autosomal recessive diseases. However, retinoblastoma is inherited in an autosomal dominant manner and thus wasn’t expected to be increased from consanguineous marriage. As a matter of fact, ASR values closer to the lower margin of reported numbers worldwide for retinoblastoma were found in this study. Regardless, genetic counseling for retinoblastoma is of crucial importance and should take place with the family of a diagnosed child soon after the diagnosis is made and with the survivor himself (the patient) when in reproductive age [1].

Cancer in the pediatric population

In this nine year cohort study in Lebanon, leukemia and myeloproliferative diseases ranked first in the pediatric population (679 cases), followed by lymphoma and reticuloendothelial neoplasms, which ranked second with 576 cases. Retinoblastoma ranked 11^th^ (38 cases), followed only by hepatic tumors (15 cases). These numbers are consistent with the American Society of Clinical Ophthalmology (ASCO) data, where leukemia accounts for 29% of childhood cancer cases while retinoblastoma accounts for only 2% [15]. We conclude that retinoblastoma is a rare diagnosis, which means that it can often be missed by primary care physicians (PCPs), especially that the signs of retinoblastoma (strabismus, leukocoria) are first noticed by parents, not by PCPs [16]. The consequences of a delayed retinoblastoma diagnosis can be disastrous; children with any cancer have a decrease in physical, social, and especially emotional health-related quality of life (HRQoL) when compared to healthy children [17]. Also, patients' siblings have a worse relationship with their family than the patients themselves.

When it comes to retinoblastoma, delayed diagnosis is feared because enucleation of the affected eye(s) might be needed with the advanced disease, which would lead to disfigurement. In reality, retinoblastoma patients seem to have a similar psychosocial functioning when compared to non-cancerous patients; this could be explained by the better prognosis of this cancer when treated early [18].

Distribution of retinoblastoma cases according to age

Out of the 38 cases of retinoblastoma in Lebanon between 2005 and 2012 and in 2015, 29 cases were in the 0-4 age group (Table 1). Compellingly, the number of cases decreased in higher age groups as no cases were recorded for the 15-19 age group. According to the American Academy of Pediatrics and the Journal of Pediatrics, most patients have retinoblastoma present before the age of five (80% even before three years of age), and the median age of diagnosis is 18 months [19]. Retinoblastoma is rare after the age of six years [20].

Variation in age-adjusted rates further confirms this point: the highest rate belongs to the 0-4 age group (7.62 cases per million person-years), the peak being in 2010 with 16.18 cases per million person-years (Table 2). In total, the age-standardized rate (ASR) was 3.61 and 2.8 cases per million person-years for the 0-14 and 0-19 age groups, respectively.

Gender distribution of cases

In the 0-4 age group, which is the age group that bears the most cases, ASR is the same for both sexes (3.64 cases per million person-years) (Table 3). The difference resides in the 5-9 age group: boys were not found to have retinoblastoma in this age group, whereas girls had an ASR of 2.32 per million person-years (Table 3). Results are congruent with other countries, where most of the retinoblastoma cases have occurred in the 0-5 age group (Table 4) [8], indicating that retinoblastoma appears in the early years of life [4]. For instance, Iran, Mauritius, and Qatar did not present any retinoblastoma cases in the 5-9 age group (Table 4).

Overall, the male/female ratio was 0.57, showing that RB was nearly twice in girls than in boys in Lebanon during the cohort. Compared to other regional Arab countries, Bahrain and Jordan were found to have a male predominance for the 0-19 age group (Table 4).

Interestingly, an overview of the genetics of retinoblastoma would account for even distribution between males and females since the only known cause of this malignancy is a mutation in the tumor suppressor gene RB1 found on chromosome 13 affected initially either by a hereditary or a sporadic mutation. In the hereditary form, the RB1 gene mutation is present in all cells, including reproductive cells; thus, there is a 50% chance to pass this mutation to the next generations (germline mutations). On the contrary, sporadic (non-hereditary) form only occurs in retinal cells; consequently, it cannot be transmitted to the next generation [1].

Thus, the gender disparities mentioned above cannot be attributed to retinoblastoma genetics but rather to the population size: all the countries listed as examples have a population not exceeding 10 million persons.

In countries with bigger sample sizes, such as the USA, the UK, and Canada, retinoblastoma had an equal sex distribution among males and females (Table 4).

Retinoblastoma in Lebanon compared to other countries

When comparing retinoblastoma in Lebanon to other regional countries, mainly the MENA region, we realize that Lebanon’s ASRs are in the median range for both 0-14 and 0-19 age groups. Morocco, a North African country, recorded the highest ASRs in the region, the second highest worldwide, with 9.2 and 7.1 cases per million person-years for the 0-14 and 0-19 age groups, respectively. Jordan came 2^nd^ with 7.1 and 5.1 cases per million person-years, respectively. The lowest ASRs recorded in both age groups (0-14 and 0-19) were in Qatar, a country in the Arabian Peninsula, with 0.4 and 0.3 cases per million person-years, respectively. Tunisia recorded the same ASRs as Lebanon, with 3.6 and 2.8 cases per million person-years for the 0-14 and 0-19 age groups, respectively (Table 4).

Compared to other randomly selected countries worldwide, Lebanon’s ASRs could be considered slightly below average for both age groups. The highest ASR recorded was for Mali, an African country, with 28.7 and 22.2 cases per million person-years for the 0-14 and the 0-19 age groups, respectively. Interestingly, we realize that African countries have higher ASRs than countries in the rest of the world. Other African countries with high ASRs further support this assumption include Morocco (previously discussed), Kenya, and Libya, among others.

We conclude that Lebanon has lower ASRs when compared to countries from other regions of the world in Europe, America (North and South), and Africa. Examples from the developed world countries include the US with 4.9 and 3.8 cases per million person-years and the UK with 4.5 and 3.5 cases per million person-years.

In an attempt to interpret such disparities between the different listed countries, risk factors for having a child with retinoblastoma were reviewed, but evidence supporting these findings was sparse. These risk factors included: diets low in fruits and vegetables during pregnancy, exposure of pregnant mothers to chemicals in gasoline, exposure of fathers to radiation, and older age among fathers [21].

The fictional population

It is primordial to acknowledge that the Syrian/Palestinian refugees constitute a vast proportion of the residents in Lebanon. According to a United Nations High Commissioner for Refugees (UNHCR) survey, 1.17 million Syrian refugees were registered with UNHCR in Lebanon (as of July 2015) [22]. This survey demonstrated that Syrian refugees were less likely to seek or receive healthcare than the local Lebanese population. As a result, we found it necessary to utilize a fictional rate where we used the Lebanese population as the denominator, excluding Syrian and Palestinian refugees. When doing so, the ASR notably differed in the 0-19 age group (increased from 2.8 to 5.16 cases per million person-years) (Table 2). Using this fictional rate, Lebanon’s ASR became above average, exceeding most regional and worldwide countries. Thus, it is primordial to shed light on the enormous degree of underdiagnosis in the refugee population and its causes, the most important one being the financial barrier [22].

Retinoblastoma trends dynamics

Using regular linear analysis, we found that retinoblastoma cases were decreasing at a rate of 22.67%. Nonetheless, this decrease is not statistically significant. Interestingly, when using joinpoint analysis, two segments were generated. The first segment ranged from 2005 to 2011, revealed a significant decline in the number of cases, and was followed by an abrupt increase between 2011 and 2015 (Figure 2B).

However, retinoblastoma is still a rare neoplasm, and resources have to be employed for appropriate detection and treatment in developing countries such as Lebanon and other Arab countries. In an effort to improve retinoblastoma healthcare in Lebanon and to be able to afford expensive equipment, the Children’s Cancer Institute at the American University of Beirut Medical Center in Lebanon allocated resources in 2012 to mount a referral center for retinoblastoma and to attract retinoblastoma cases from Iraq and Syria in an attempt for efficient interdisciplinary coordination [23]. 

This study has several limitations, the most important one being the missing data from 2013 and 2014 and the publicly unavailable data for retinoblastoma genders for most of the years. Furthermore, data for retinoblastoma is also unavailable for the years after 2015.

## Conclusions

In conclusion, as Lebanon harbors both diseases of affluent countries and diseases of developing countries, the numbers for retinoblastoma are in the lower margin of the average recorded worldwide, witnessing an increase in the number of cases from 2011-2015. Underdiagnosis in the refugee population might be underestimating the prevalence of retinoblastoma in Lebanon. Efforts are being deployed to overcome the financial barriers in the treatment of retinoblastoma by coordinating with neighboring Arab countries and acknowledging the importance of early diagnosis and early treatment.
